# Age-related changes in mechanical properties of semitendinosus tendon used for anterior cruciate ligament reconstruction

**DOI:** 10.1186/s13018-022-03395-9

**Published:** 2022-11-19

**Authors:** Takuto Akazawa, Naokazu Miyamoto, Hirofumi Nishio, Eri Miyamoto-Mikami, Mayuko Kinoshita, Yohei Kobayashi, Masashi Nagao, Yuji Takazawa

**Affiliations:** 1 Graduate School of Health and Sports Science, Juntendo University, 1-1 Hiraka-Gakuendai, Inzai, Chiba 270-1695 Japan; 2 Department of Sport Science and Research, Japan Institute of Sports Sciences, Tokyo, 3-15-1 Nishigaoka, Kita-ku, Tokyo 115-0056 Japan; 3 Department of Orthopaedic and Motor Organ, Faculty of Medicine, Juntendo University, 2-1-1, Hongo, Bunkyo-ku, Tokyo 113-8421 Japan; 4 Department of Orthopaedic Surgery, Oji Hospital, 2-14-13, Oji, Kita-ku, Tokyo 114-0002 Japan; 5 Medical Technology Innovation Center, Juntendo University, 2-1-1, Hongo, Bunkyo-ku, Tokyo 113-8421 Japan

**Keywords:** Young’s modulus, Elongation, Creep, Tensile testing, Hamstring, Autograft

## Abstract

**Background:**

Hamstring tendons are a popular choice for autografts in anterior cruciate ligament (ACL) reconstruction. However, there is increasing evidence that hamstring tendon autografts carry a high risk of revision and residual instability in young patients. To elucidate the reasons for the inferior outcome of the reconstructed ACL with hamstring tendon autografts in young patients, we investigated the Young’s modulus and the extent of cyclic loading-induced slackening of the semitendinosus tendon used for ACL reconstruction across a broad range of ages.

**Methods:**

Twenty-six male patients (aged 17–53 years), who were scheduled for ACL reconstruction surgery using the semitendinosus tendon autograft, participated in this study. The distal portion of the harvested semitendinosus tendon, which was not used to construct the autograft, was used for cyclic tensile testing to calculate the Young’s modulus and the extent of slackening (i.e., increase in slack length).

**Results:**

Spearman correlation analysis revealed that the Young’s modulus of the semitendinosus tendon was positively correlated with the patient’s age (*ρ* = 0.559, *P* = 0.003). In contrast, the extent of tendon slackening did not correlate with the patient’s age.

**Conclusions:**

We demonstrated that the Young’s modulus of the semitendinosus tendon increases with age, indicating that the semitendinosus tendon used for ACL reconstruction is compliant in young patients.

## Background

The anterior cruciate ligament (ACL) is the most commonly injured knee ligament, and 175,000–200,000 patients undergo ACL reconstruction surgery every year in the USA alone [1]. Hamstring tendons, primarily using the semitendinosus (ST) tendon, with additional gracilis tendon if necessary, are a popular choice for autografts in ACL reconstruction, owing to their easy harvesting technique, low harvest site morbidity, and mechanical superiority [2, 3]. ACL reconstruction with a hamstring tendon autograft is usually successful and satisfactory. However, there is increasing evidence that hamstring tendon autografts carry high risks of revision and residual instability in young patients [4–9]. For example, patients aged less than 20 years [4] or 25 years [5] are shown to be at risk of revision after ACL reconstruction using the hamstring tendon. In addition, a recent systematic review reported that young age is the most consistent factor for failure of ACL reconstruction [6]. It has been suggested that higher failure rates in younger patients could be attributable to higher postoperative active levels [7], while the risk of hamstring autograft failure is suggested to be independent of individual’s activity levels [5]. Taken together, it remains unclear why young patients undergoing ACL reconstruction with hamstring tendon autografts show an inferior outcome.

One of the reasons for the inferior outcome of ACL reconstruction in young patients would be associated with the age-related difference in the mechanical properties of the graft used for ACL reconstruction. Several in vivo human studies have compared the mechanical properties of tendons, including the Achilles [10] and patellar tendons [11, 12], between children (approximately 10–18 years old) and young adults, and reported that the Young’s modulus (a fundamental parameter that characterizes the stiffness of a material, expressed in Pascal) of the tendon was lower in children than in adults. In contrast, it has been recently reported that the Young’s modulus of hamstring tendons harvested during ACL reconstruction is significantly higher in younger patients (≤ 20 years) than older patients (> 20 years) [13]. The discrepant findings between the previous studies are attributable at least partly to the differences in tendons tested [14] and the sex of the subjects [15]. Particularly regarding the latter, age-related changes in tendon mechanical properties have been reported to vary between males and females [15]. Nevertheless, in the previous study [13], the Young’s modulus of hamstring tendons harvested during ACL reconstruction has been examined in a mixed population of males and females. An advanced understanding of the age-related changes in the Young’s modulus of the hamstring tendon used for ACL reconstruction in a single-sex population would allow for more effective surgical treatment and rehabilitation.

Several studies have indicated that the elongation of the graft used for ACL reconstruction occurs without trauma [8, 16]. To prevent such secondary graft elongation, pre-tensioning of the grafts before fixation has been recommended [17, 18]. Meanwhile, patients aged < 20 years exhibited greater postoperative knee joint laxity, probably due to graft elongation, than those aged ≥ 20 years [19]. Based on this finding, it is presumed that the ST tendon used for ACL reconstruction is more susceptible to slackening in young patients, leading to an inferior outcome of ACL reconstruction. To our knowledge, however, no studies have examined the association between patient age and susceptibility to slackening of the ST tendon used for ACL reconstruction. More specifically, although several studies emphasize the importance of cyclic tensile (i.e., repeated loading–unloading) testing when evaluating the mechanical properties of tendons [20], single load-to-failure testing has most often been used; no information is available on whether susceptibility to cyclic loading-induced tendon slackening is age-dependent.

As a first step to elucidate the reasons for the inferior outcome of the reconstructed ACL in young patients, we compared and investigated the Young’s modulus and the extent of cyclic-loading-induced slackening of the ST tendon used for ACL reconstruction across a broad range of ages in a single-sex population of males. We hypothesized that the Young’s modulus and the extent of slackening of the ST tendon used for ACL reconstruction would be smaller and greater in younger than older patients, respectively.

## Methods

### Subjects

Twenty-six male patients (aged 17–53 years), who were healthy except for an ACL injury and scheduled for ACL reconstruction surgery with ST tendon autograft, participated in this study. The present study introduced no selection bias in sample collection; patients who were scheduled for ACL reconstruction surgery with ST tendon autograft and agreed to participate in the present study were included. All subjects provided written informed consent prior to participation. This study was approved by our institutional ethics committee.

### Preparation of specimens

**Fig. 1:**
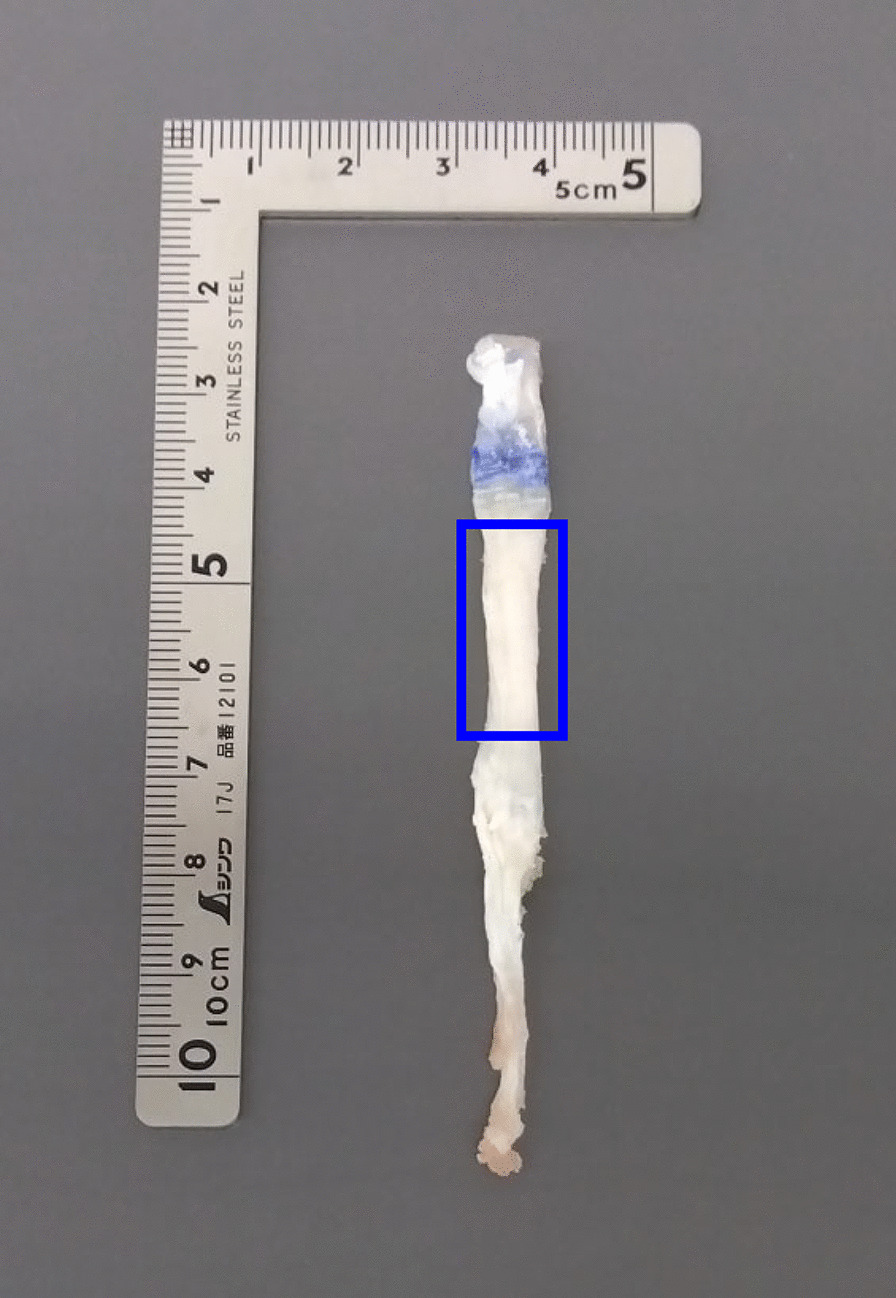
Typical example of the distal portion of the harvested semitendinosus tendon specimen. A uniform thickness portion of the specimen harvested (as outlined by the solid blue line) was prepared rectangularly (i.e., uniform in width and length) for tensile testing

The ST tendon was harvested with a standard technique using a tendon stripper after making a 3-cm skin incision to identify the ST tendon attachment during ACL reconstruction surgery. The distal portion of the harvested ST tendon (Fig. 1), which was discarded and not used to construct the autograft, was embedded in phosphate-buffered saline and stored at − 20 °C until the time of mechanical tensile testing.

### Measurement

**Fig. 2:**
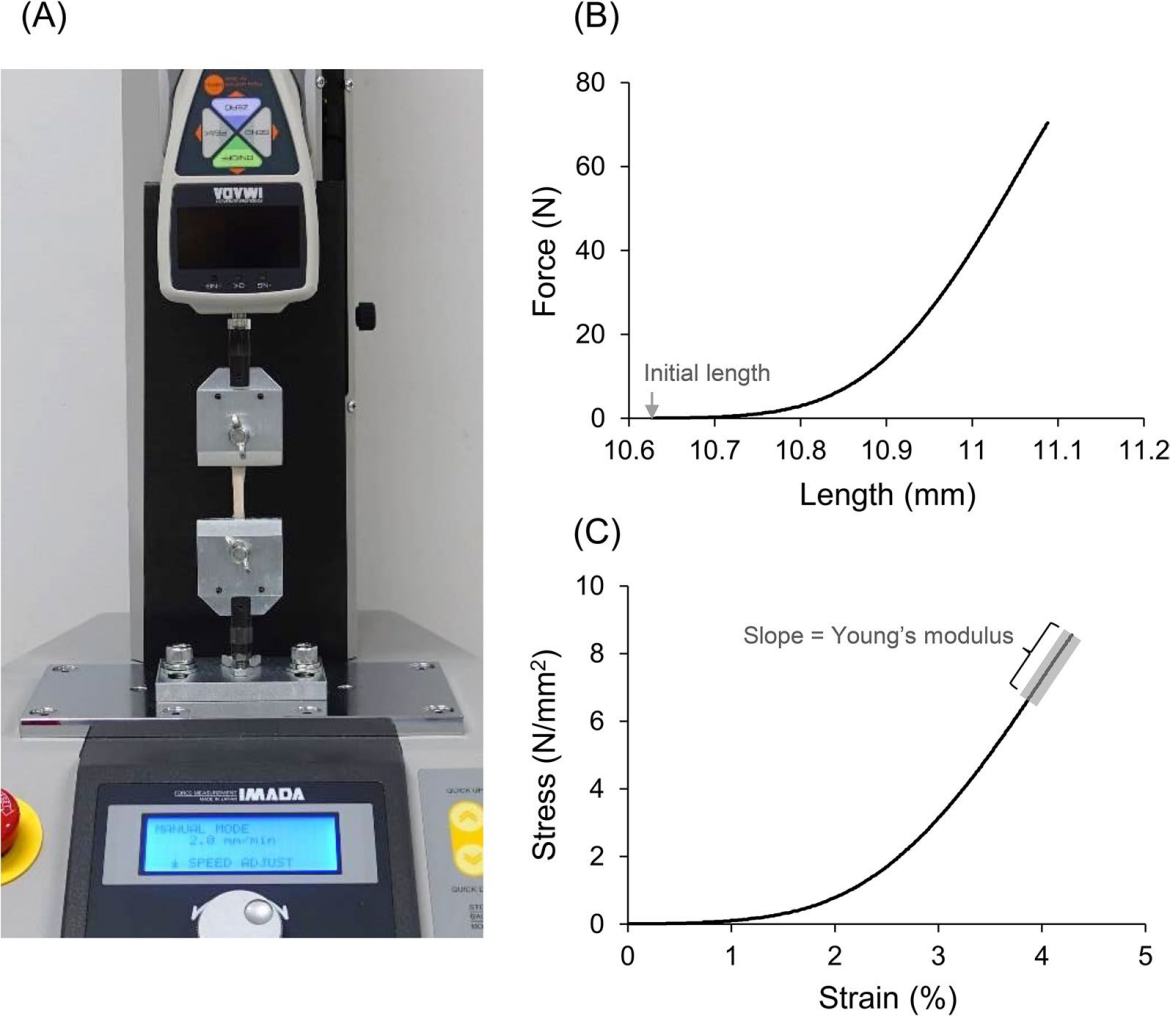
Typical examples of experimental setup (**A**) and data analysis process (**B**, **C**) for tensile testing. The ends of the tendon specimens were fixed to the clamps of material testing machine (**A**). Force and length were measured by tensile testing, and a force–length relationship was derived (**B**). The stress (N/mm^2^) was calculated by dividing the force by the cross-sectional area of the specimen, and the strain (%) was calculated by dividing the displacement (i.e., change in length) of the specimen by its initial length (**C**). The Young’s modulus of each specimen was calculated as the slope of the regression line in the linear region of the stress–strain curve

On the day of mechanical testing, the tendon specimens were first thawed at room temperature and then cleaned by removing the muscle and fatty tissues. Then, uniform-thickness portions of the specimens harvested (Fig. 1) were prepared rectangularly (i.e., uniform in width and length) for tensile testing. The lengths of the specimens shaped for tensile testing were approximately 15–20 mm. In our preliminary experiments, specimens were attached to the clamps of a material testing machine using sandpapers, according to previous studies [21]. However, substantial slippage between the clamps and sandpapers or between the sandpapers and specimens was often observed during tensile tests. In the present study, therefore, to prevent the slippage during the tensile test, both ends (approximately 3–4 mm each) of the tendon specimens were directly fixed to the clamps (FC-40-F, IMADA, Japan) of a material testing machine (EMX-1000N, IMADA, Japan) (Fig. 2A) using cyanoacrylate. Each specimen was subjected to 10 loading–unloading cycles [20] from the slack state at a speed of 2 mm/min [22]. The loading displacement was not uniform across specimens but was dependent on the stiffness of the specimen in the linear region. The loading force was measured using a force gauge (ZTA-200A, IMADA, Japan) mounted between the material testing machine and the clamp. The force and length (i.e., clamp-to-clamp distance) data were recorded at a frequency of 100 Hz (Fig. 2B). The experimenters visually confirmed that no slippage occurred during the tensile tests. These measurements were performed at a room temperature of approximately 23 °C. After the completion of the tensile test, a cross-section at the approximate midpoint of each specimen was prepared by cutting it perpendicular to the tensile direction using a microtome blade (Surgipath DB80 HS, Leica Biosystems, Germany). Then, the cross-section was captured using a high-resolution (4K) digital camera (RX100IV, Sony, Japan).

### Data analysis

For the mechanical testing data, the stress (N/mm^2^) was calculated by dividing the force by the cross-sectional area (CSA) of the specimen, and the strain (%) was calculated for each cycle by dividing the displacement (i.e., change in length) of the specimen by its initial length (Fig. 2C). The CSA of the tendon specimen was calculated using publicly available software (ImageJ, NIH, USA), and the average of the three CSA measurements was used as the representative value for each specimen. The initial length was defined as the length at the point in which a 0.1 N load was detected in each cycle (Fig. 2B). The mean initial length of the ST tendon specimens tested was 8 mm. The Young’s modulus of each specimen was also calculated for each cycle as the slope of the regression line in the linear region (the final 20% stress) of the stress–strain curve (Fig. 2C). The extent of slackening from the 2nd to the 10th cycles of each specimen was calculated according to the following equation: the extent of slackening (%) = (initial length of respective cycle − initial length of the 1st cycle)/initial length of the 1st cycle × 100. For the Young’s modulus and extent of slackening of each specimen, the average values from the 6th to the 10th cycles were used as the representative values to avoid a conditioning effect of the loading–unloading cycle on the mechanical properties of tendinous tissues [23]. However, note that the calculated extent of slackening is affected by the possible conditioning effect because the initial length of the 1st cycle is used to calculate the extent of slackening, as mentioned above. Thus, to evaluate the extent of slackening that was not affected by the possible conditioning effect, the extent of slackening was calculated based on the initial length of the 6th cycle, i.e., the extent of slackening of the 7th–10th cycles of each specimen was also calculated by the following equation: the extent of slackening (%) = (initial length of respective cycle − initial length of the 6th cycle)/initial length of the 6th cycle × 100. The average extent of slackening from the 7th to 10th cycles of each specimen was used for statistical analyses.

### Statistical analysis

There were no direct data from previous studies to be referred to for a priori power analysis because none has examined the association between patient age and mechanical properties of the ST tendon across a broad range of ages. Consequently, the minimum sample size capable of detecting a statistically significant correlation was calculated with an inferred type 1 error of 0.05, a statistical power of 0.80 (type 2 error rate of 0.2), and a large effect size (0.5) using G*Power 3.1.9.4 (Kiel University, Germany). The required sample size was estimated to be 26.

The Shapiro–Wilk test did not show a normal distribution for the patient’s age (*P* = 0.003) and ST tendon Young’s modulus (*P* < 0.001). Correlations between variables were examined using nonparametric Spearman correlation coefficients. When appropriate, unequal variance unpaired t test was performed to compare the variables between the patients under and over 20 years of age. All data are reported as the mean ± SD. The significance level for all comparisons was set at *P* = 0.05.

## Results

**Fig. 3:**
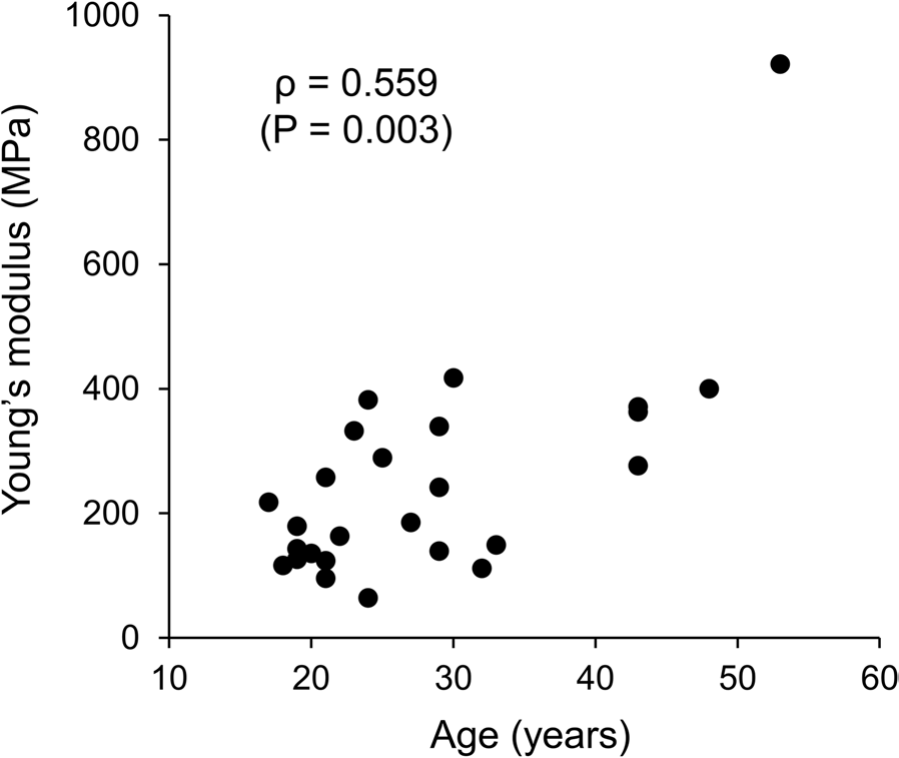
Relationship between the Young’s modulus of the semitendinosus tendon specimen and patient age. The Young’s modulus of the ST tendon specimens was positively correlated with the patient’s age (*n* = 26). Note that the correlation remained significant even if an outlier-like data from the 53-year-old subject was removed (*n* = 25)

**Fig. 4:**
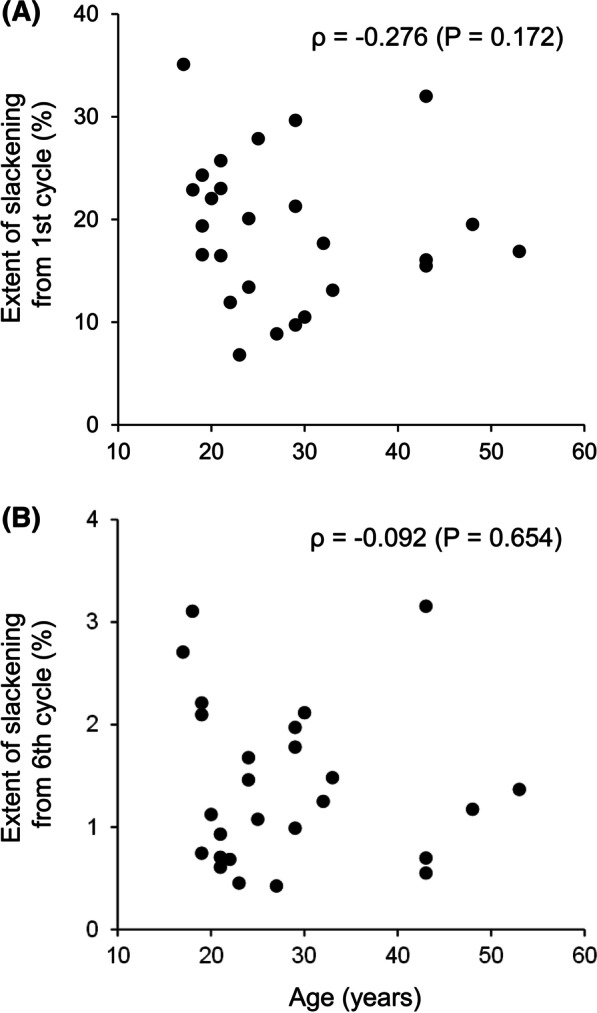
Relationship between the extent of slackening of the semitendinosus tendon specimen and patient age. The extent of slackening was calculated based on the initial length of the 1st (**A**) or 6th cycle (**B**). The extent of slackening from both the 1st and 6th cycles was not significantly correlated with patient age

The characteristics of the subjects are presented in Table 1. The Young’s modulus of the ST tendon specimens was positively correlated with the patient’s age (*ρ* = 0.559, *P* = 0.003; Fig. 3). Although this significant correlation may seem to be due to the outlier-like data from the 53-year-old subject, the correlation remained significant even if the data were removed (*n* = 25, *ρ* = 0.504, *P* = 0.010). In contrast, the Young’s modulus of the ST tendon specimens was not significantly correlated with the CSA (*ρ* =  − 0.214, *P* = 0.295) or the initial length of the specimen (*ρ* =  − 0.071, *P* = 0.729). The unequal variance unpaired t test revealed a significant in the Young’s modulus difference between the patients under and over 20 years of age (under 20: 153.3 ± 38.4 MPa, over 20: 281.5 ± 188.1 MPa; *P* = 0.009). The extent of slackening from both the 1st and 6th cycles was not significantly correlated with patient age (Fig. 4). Physical characteristics other than the patient’s age (i.e., height, body weight, body mass index) were not significantly corrected with the Young’s modulus or the extent of slackening (Table 2).

**Table 1:** Characteristics of subjects

Age (years)	28.2 ± 10.1 (17–53)
Height (cm)	174.4 ± 7.3 (162–191)
Body weight (kg)	75.9 ± 11.5 (60–101)
BMI (kg/m^2^)	25.0 ± 3.3 (20.1–33.6)

Data are shown as mean ± standard deviation

The ranges of values are shown in parentheses

*BMI* Body mass index

**Table 2:** Correlation coefficients between physical characteristics and semitendinosus tendon properties

	Young’s modulus	Slackening from 1st cycle	Slackening from 6th cycle
Age	0.559	− 0.276	− 0.092
	(*P* = 0.003)	(*P* = 0.172)	(*P* = 0.654)
Height	− 0.028	0.170	− 0.054
	(*P* = 0.898)	(*P* = 0.439)	(*P* = 0.808)
Body weight	− 0.020	− 0.132	− 0.227
	(*P* = 0.927)	(*P* = 0.550)	(*P* = 0.298)
BMI	0.063	− 0.136	0.109
	(*P* = 0.774)	(*P* = 0.535)	(*P* = 0.621)

Correlation coefficients were calculated using Spearman’s *ρ* (*n* = 26). *P* values are shown in parentheses

*BMI* Body mass index

## Discussion

To the best of our knowledge, this is the first study to investigate the correlation between mechanical properties (the Young’s modulus and the extent of slackening) of the ST tendon used for ACL reconstruction and age across a broad range of ages in a single-sex population of males. One of the greatest strengths of the present study is that the tendon samples were obtained from patients who underwent ACL reconstruction surgery. The main finding of the present study was that the Young’s modulus of the ST tendon specimen was positively correlated with the patient’s age, while the extent of slackening was not correlated with age. These findings may have important practical implications for improved surgical treatments.

The present study showed that the Young’s modulus of the tendon was positively related to age, which supports our hypothesis and contradicts the previous finding that the Young’s modulus of hamstring tendons harvested during ACL reconstruction is significantly higher in younger patients (≤ 20 years) than older patients (> 20 years) [13]. As stated in the earlier part of this paper, the discrepancy is likely due to the difference in sex of the subjects [15]. Seven males and 13 females were mixed as the subjects in the previous study, while only males were included in the present study. Our finding is consistent with previous animal [24, 25] and in vivo human studies [10–12] showing that the Young’s modulus of the tendinous tissues increases with increasing age, and the tendon Young’s modulus reported in the present study was within the range of in vivo human studies [10–12, 24, 25]. Age-related changes in the mechanical properties of tendons are reportedly associated with the collagen fibril diameter [26]. A recent study demonstrated that the cell number and collagen fibrils diameter of the ST tendon used for ACL reconstruction were different among immature (aged 10.1 ± 1.6 years), young (aged 16.7 ± 1.8 years), and adult (aged 34.6 ± 9.2 years) patients [27]. Moreover, previous studies have shown that cross-linking and glycation of tendon collagen have an impact on the Young’s modulus of the tendon, and the levels of cross-linking and glycation change with age [28–30]. Furthermore, it is possible that age-related variations in tendon mechanical properties are related, at least in part, to a loss of regenerative potential of the hamstring tendons with aging [31–33].

ACL reconstruction with a hamstring tendon autograft is at a high risk of failure or rupture in young patients [4–9]. A recent systematic review suggested that the hamstring tendon autograft diameter should be > 7 mm to prevent hamstring tendon autograft failure or rupture [9]. In contrast, another study showed a high risk of graft failure in rugby players aged < 20 years than in those aged ≥ 20 years, despite the fact that all hamstring tendon autograft diameters were greater than 7.5 mm [8]. The reason for the higher risk in younger patients despite the use of larger diameter grafts than recommended by the systematic review [9] may be related to the lower Young’s modulus of the graft in younger patients, as shown in this study. Therefore, to reduce the force applied per graft CSA and thereby reduce the displacement (i.e., change in length) of the graft under a given force, hamstring tendon autografts with a larger diameter may be helpful in improving the outcomes of ACL reconstruction with hamstring tendon autografts for younger patients. Otherwise, from the mechanical point of view, for materials with the same Young’s modulus, longer ones exhibit large displacement against a given force than shorter ones. Thus, to decrease the displacement of grafts under a given force, a graft shorter than usual may be useful in young patients.

Contrary to our hypothesis, we failed to demonstrate a significant age dependence of the extent of cyclic loading-induced slackening. Based on the findings of the present study, the greater postoperative knee joint laxity in patients aged < 20 years observed in a previous study [19] seems to be not due to greater susceptibility to slackening of the ST tendon in younger patients. Before drawing a general conclusion from this finding, however, we should note that this finding is based on 10 cycles of loading–unloading induced under ex vivo conditions and that much more loading–unloading induced under in vivo conditions may have led to different results. Nevertheless, in the present study, most of the extent of slackening occurred in the first three cycles, while the extent of slackening caused by the sixth and subsequent cycles was much smaller. Therefore, the effect of the number of loading–unloading cycles on the present findings would be small.

The present study has some limitations. First, for technical reasons, we could not measure the mechanical properties of the ST tendons immediately after harvesting. To minimize biological degradation, the tendons were stored at − 20 °C. Goh et al. [34] showed that freezing at − 20 °C had no effect on the mechanical properties of animal tendons, including the Young’s modulus, while freezing at − 80 °C led to an increase in the Young’s modulus. Second, all subjects were male. The mechanical properties of tendons differ according to sex [35]. Additionally, it has been indicated that female hormones influence the mechanical and structural properties and collagen synthesis of tendons [36, 37] and that the adaptability of tendons differs by sex [38]. Indeed, age-related changes in tendon mechanical properties have been reported to vary between males and females [15]. Thus, the present findings may not be valid for female patients. Third, the distal portion of the harvested ST tendon was used in this study. Although a cadaver study reported no significant difference in mechanical properties (Young’s modulus, ultimate stress, ultimate strain, and strain energy density) between the proximal and distal portions of the ST tendon [39], it is unknown whether the tendon specimens used in the present study represent the whole ST tendon used for ACL reconstruction. Fourth, the minimum age of the subjects in the present study was 17 years, and only five were under 20 years old. Therefore, it is practically impossible to determine from the current data whether and how the Young’s modulus of the ST tendon increases with age in young patients (i.e., those under 20 years). Further studies are required to examine these points.

## Conclusion

We provide evidence that the Young’s modulus, but not the extent of slackening, increases with age. This indicates that the semitendinosus tendon used for ACL reconstruction is compliant in young patients. The findings obtained here may lead to an improved understanding of ACL reconstruction surgery.

## Data Availability

The datasets used and/or analyzed during the current study are available from the corresponding author on reasonable request.

## Abbreviations

ACL: Anterior cruciate ligament
ST: Semitendinosus
CSA: Cross-sectional area
